# Personal recovery in mental health difficulties in people with experience of homelessness: qualitative systematic review

**DOI:** 10.1192/bjo.2025.10851

**Published:** 2025-11-04

**Authors:** Jessica A. E. Dring, Rosie Powell Davies, Neil Carrigan

**Affiliations:** Oxford Institute for Clinical Psychology Training and Research, University of Oxford, Isis Education Centre, Warneford Hospital, UK

**Keywords:** Homelessness, qualitative research, personal recovery, mental health services, CHIME

## Abstract

**Background:**

Given the complex challenges facing people experiencing homelessness, existing mental health recovery models are probably insufficient for this population.

**Aims:**

To investigate qualitative accounts of mental health personal recovery in people with experience of homelessness, and to adapt the widely adopted connectedness, hope, identity, meaning and empowerment (CHIME) model of personal recovery to better represent the experiences of this population.

**Method:**

PROSPERO registration no. CRD42023366842. A systematic review identified qualitative studies investigating first-person accounts of mental health personal recovery in people with experience of homelessness. Nine databases were searched: CINAHL, SCOPUS, Embase, Medline, PsychINFO, PubMed, Web of Science, ASSIA and Social Services Abstracts. Risk of bias was assessed using the Critical Appraisal Skills Programme (CASP) Qualitative Studies Checklist. Included studies underwent ‘best fit’ framework synthesis, comprising deductive analysis using the CHIME first- and second-order themes, as well as inductive analysis to capture aspects not covered by the *a priori* framework.

**Results:**

The review expanded the CHIME model and identified the following recovery processes in this population: security and stability; encouragement and hope; constructing identity; understanding and meaning; relationships and connectedness; and empowerment and dual recovery (SECURED). Importantly, security and stability were identified as a necessary prerequisite for the other recovery processes. Challenges within each recovery process were also identified.

**Conclusions:**

SECURED offers a transdiagnostic framework to support understanding of mental health personal recovery in the context of homelessness. Findings support the Housing First model of service provision. However, findings also highlight that housing alone is not sufficient and that the other processes must also be supported.

The notion of recovery is central to mental health policy and research.^1–3^ However, there is no universal definition of recovery. Contention exists between conceptualisations of ‘recovery from’ versus ‘recovery in’ mental health difficulties. The former takes a clinical approach, referring to a remission of symptoms.^4^ The latter is labelled personal recovery, expert-by-experience led and refers to individuals living meaningful and satisfying lives despite potential ongoing mental health difficulties.^4^ Research synthesising lived experience accounts of recovery favour the personal recovery conceptualisation, emphasising its individual nature, but reveal some universal components of recovery.^5,6^ Identifying common components in the lived experience of recovery can aid the configuration of services to better enable meaningful recovery-orientated delivery.^5^ Leamy and colleagues^7^ conducted a large-scale systematic review which synthesised qualitative research that described or developed conceptualisations of personal recovery, to develop the connectedness, hope, identity, meaning and empowerment (CHIME) model of personal recovery. The CHIME model comprises five themes: connectedness (support, relationships and community); hope (optimism, aspirations and motivation); identity (positive identity and overcoming stigma); meaning (meaningful roles and activities, quality of life and spirituality); and empowerment (goals and focusing on strengths). The CHIME model has been widely implemented in mental health services and training to promote recovery-oriented practices,^8^ and has been applied to randomised controlled trials of recovery-oriented intervention^9,10^ and personal recovery measures.^11,12^ However, it has been highlighted that CHIME neglects issues of culture and difficulties inherent to recovery, such as trauma, victimisation, stigma and negative life changes.^13,14^ Thus, it is important that the CHIME model be adapted to distinct population characteristics.^14^

The research into mental health recovery that informed the CHIME model has largely overlooked the effects of poverty, discrimination and exclusion, particularly homelessness.^15,16^ The prevalence of mental health difficulties is higher in those experiencing homelessness compared with the general population, with research suggesting rates of 58–100% in European countries.^17^ Padgett and colleagues^15^ coined the term ‘complex recovery’ to better capture overcoming multiple forms of adversity in those who have experienced homelessness. Individuals experiencing homelessness may be faced not only with mental health difficulties but also stigma, poverty, unemployment, chronic medical conditions, housing issues, trauma histories, victimisation, effects of prior incarceration, social exclusion and many other adversities.^18,19^ Accordingly, the CHIME model probably does not fully capture the experiences of personal recovery in people with experience of homelessness. Nevertheless, research shows that positive changes are possible for those experiencing homelessness regarding mental health, physical health, substance misuse and social integration.^20–22^ However, less is known about the recovery processes that underlie positive changes in individuals with experience of homelessness and mental health difficulties.^19^

There is growing research into mental health recovery in those experiencing homelessness, with some highlighting the usefulness in taking a personal recovery-orientated approach within the homeless service system.^22^ Given the unique and complex challenges facing the homeless population, existing recovery models such as CHIME may not capture the perspectives of personal recovery in individuals with experience of homelessness.^7^ Qualitative research into the perspectives of recovery in people experiencing homelessness and mental health difficulties can offer valuable insight into the recovery processes of this population.^23^ However, there is currently no systematic review or framework that synthesises this research. Such a framework would be useful in informing clinical understanding and practice. Therefore, the aims of the current project are to systematically review the qualitative research into the experiences of mental health personal recovery in individuals with experience of homelessness. Through this, the review also aims to adapt the CHIME model of recovery for those with experience of homelessness.

## Research questions

The current study aimed to answer the following questions:

- What does qualitative research reveal about mental health personal recovery in people with experience of homelessness?
- Based on the above, does the CHIME recovery model need to be adapted to account for the experiences of mental health personal recovery in people with experience of homelessness?

## Method

The Enhancing Transparency in Reporting the Synthesis of Qualitative Research (ENTEQ) and Preferred Reporting Items for Systematic Reviews and Meta Analyses (PRISMA) guidelines informed the methodology and reporting of this review.^24,25^ The review was registered with PROSPERO (registration no. CRD42023366842).

### Eligibility criteria

The eligibility criteria (see Table 1) were designed to identify the existing peer-reviewed published evidence base for qualitative first-person accounts of mental health personal recovery in adults with experience of homelessness, guided by ENTEQ guidelines.^24^ Due to this being an emerging area of research, the broad Public Health England definition of homelessness – being without a stable home – was used.^26^ Accordingly, this definition included rough-sleeping, staying in temporary accommodation (e.g. shelter or hostel) or sofa-surfing. The criterion of no limit on publication year allowed as broad a search as possible.

**Table 1:** Eligibility criteria

	Inclusion criteria	Exclusion criteria
Concepts	Perceptions and experiences of mental health personal recovery^a^ from a first-person perspective	Perceptions and experiences of recovery expressed by others (e.g. staff and family)
		Perceptions and experiences of substance misuse recovery only
		Evaluations of interventions regarding what was ‘good’ or ‘bad’ about specific aspects of interventions rather than what supported or underpinned personal recovery
		Barriers to recovery only
Methodology	Qualitative method for data collection and data analysis	Quantitative methodology
Population	Individuals with current or past experience of homelessness	Individuals without experience of homelessness
Age (years)	18+	Under 18
Year limits	Any time	No limit
Language	Published in English or able to be translated into English	Studies not published in English or not able to be translated into English
Article	Published in a peer-reviewed Journal	Not published in a peer-reviewed journal
	Original research article	Not an original research article, including review, report or book

a. Defined as the process of developing a meaningful and satisfying life with or without continuing symptoms.

### Search strategy

A comprehensive literature search was conducted in November 2023, and updated in April 2024, on CINAHL, SCOPUS, Embase, Medline, PsychINFO, PubMed, Web of Science, ASSIA and Social Services Abstracts. Search terms related to homelessness, personal recovery, mental health difficulties and qualitative methodology. An experienced librarian consulted on and checked the search strategy. A manual search of the reference lists of studies meeting the inclusion criteria, and a secondary search on Google Scholar, were conducted to facilitate a comprehensive search.

### Study selection

The lead author (J.A.E.D.) screened all titles and abstracts, with 20% screened by a second reviewer (R.P.D.), and a strong level of agreement was reached between the two reviewers (*k* = 0.86). Discrepancies were discussed and resolved between the reviewers. Full-text articles were assessed for eligibility by the lead author and 25% assessed separately by a second reviewer (R.P.D.); again, a strong level of agreement was reached between the two reviewers (*k* = 0.90). In May 2025, the remaining 80% of titles and abstracts were screened by a second reviewer (R.P.D.), with a strong level of agreement again reached between the two reviewers (*k* = 0.93). Discrepancies were discussed and resolved between the reviewers, and no further studies were included. In May 2025, the second reviewer (R.P.D.) also screened the remaining 75% of full-text articles, with full agreement (*k* = 1.00). Therefore, no further studies were included following the updated full-text screening.

### Data extraction

The data were extracted by the lead author, starting on 6 February 2024, with a second reviewer (R.P.D.) independently checking a sample of 25% for correctness and completeness of data. No discrepancies between the data extracted by the lead author and the sample checked by the second reviewer were found. In May 2025, a second reviewer (R.P.D.) independently extracted data for all included studies, and this was compared with the data extracted by the lead author; no discrepancies between the data extracted by the lead author and second reviewer were identified (*k* = 1.00). Study characteristics extracted included title, author, publication year, country of origin, sample size, mental health diagnoses, participants’ age, gender and ethnicity, data collection method, data analytic approach and themes.

### Study of risk of bias assessment

The quality of papers was assessed using the Critical Appraisal Skills Programme (CASP) Qualitative Studies Checklist.^27^ CASP is the most frequently used qualitative appraisal tool.^28^ The lead author reviewed all included papers and a second reviewer (R.P.D.) assessed 25% of them, with high agreement reached across the CASP rating criteria (*k* = 92). Discrepancies were resolved though discussion of rationales to reach a consensus. In May 2025 the remaining 75% of papers were assessed by a second reviewer (R.P.D.), with a high level of agreement reached across the CASP rating criteria (*k* = 94). Discrepancies were again resolved through discussion of rationales to reach a consensus.

### Data synthesis

A total of 16 papers were synthesised following the ‘best fit’ framework synthesis guidelines.^29^ The RETREAT mnemonic (review question, epistemology, time/timescale, resources, expertise, audience and purpose, and type of data) helped to guide the choice of analytical method.^30^ Best fit framework synthesis was chosen because it allows an existing model to be tested on, and/or adjusted to, a particular population or context.^29^ The method is also useful when examining a complex topic such as personal recovery, wherein theory can aid with understanding generalisable factors influencing the delivery and effectiveness of interventions and policy.^31^ CHIME was chosen as a best fit *a priori* framework because it is a well-established and endorsed model of personal recovery^14^ that has been applied to randomised controlled trials of recovery-oriented intervention^9,10^ and personal recovery measures,^11,12^ and has demonstrated good applicability as an *a priori* framework for other systematic reviews into personal recovery in specific populations.^32–34^

Data for analysis were extracted from ‘findings’ or ‘results’ sections from included studies, consisting of quotations and authors’ summaries of the findings.^35^ Extracted data were uploaded into NVivo software version 12 for Windows (Lumivero, Denver, CO, USA; see https://lumivero.com/company/). The lead author first repeatedly read the articles to familiarise themselves with the data, then completed line-by-line coding of the data for each article. This process involved both deductive and inductive coding. Data were deductively coded according to the first- and second-order themes in the CHIME model (see Table 2).^7^ For data that were not adequately captured by CHIME, inductive thematic analysis was used^36,37^ to generate new codes. Both deductive and inductive codes were grouped according to similarities and differences to form broader patterns of meaning and preliminary themes. This resulted in some of the *a priori* themes being adjusted to better reflect the data, as well as the formation of new themes. *A priori* themes were also retained or removed, subject to the richness and depth of the coded data. New and revised second-order themes were assigned to first-order themes in the CHIME model, and those that could not be accommodated were grouped to form new first-order themes. This formed a revised conceptual framework. The lead author presented the results to two experts-by-experience with lived experience of homelessness and mental health difficulties.

### Reflexivity and epistemological stance

Reflexivity is important in understanding the influences of researchers’ backgrounds and experiences in qualitative research.^38^ The lead author is a 28-year-old White British woman with an academic and clinical background in mental health as a trainee clinical psychologist within the UK National Health Service (NHS). The researchers do not have first-hand experience of homelessness. The lead author was interested in the use of qualitative research to centre first-person accounts of under-researched and disadvantaged groups in the mental health field. Reflexivity was supported by keeping a reflective journal during analysis, as well as via regular discussions regarding emerging interpretations with supervisors and consultation with experts-by-experience. Best fit framework synthesis takes a realist epistemological stance, aiming to test or adjust existing theory by exploring the underlying mechanisms that may explain a phenomenon.^39^

## Results

### Identification of studies

**Fig. 1:**
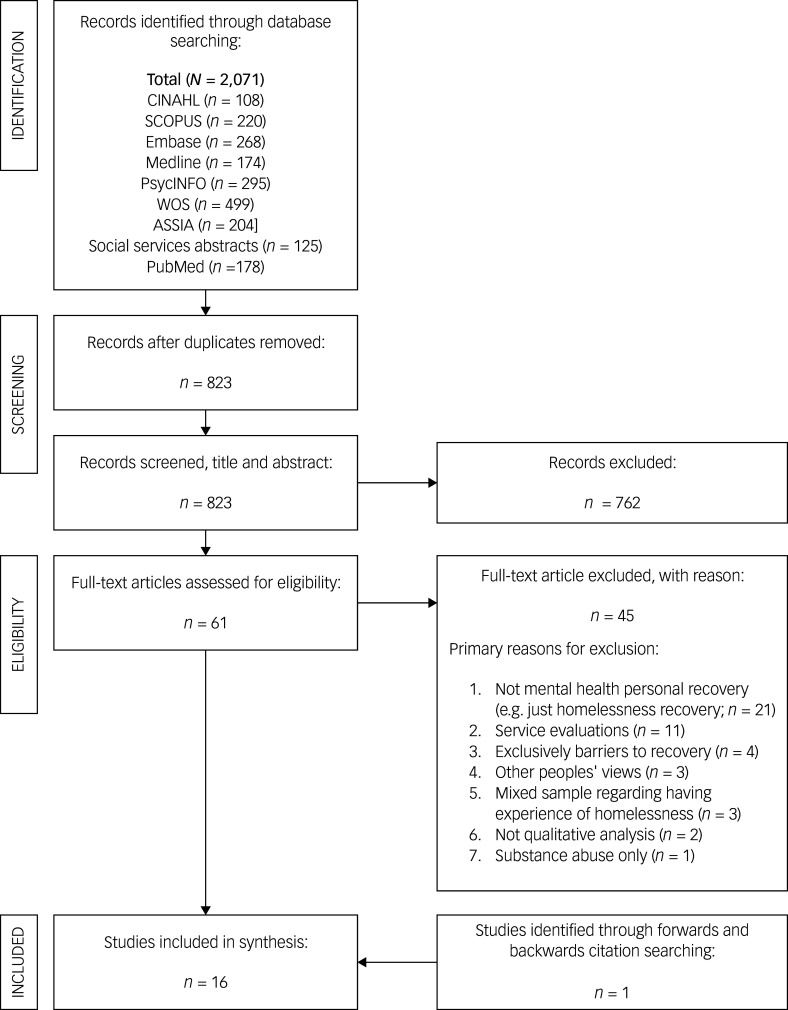
Preferred Reporting Items for Systematic Reviews and Meta Analyses (PRISMA) flowchart detailing the search strategy.

A PRISMA flow diagram illustrates the study selection process^25^ (see Fig. 1). The combined electronic database search retrieved 2071 record and, after removing duplicates, 823 articles remained. Following review of title and abstract to determine eligibility, 61 full-text articles were reviewed in full against eligibility criteria. This resulted in 16 papers being included in the qualitative synthesis.

### Study selection and demographics

The detail of sample demographic information in studies varied. The studies comprised qualitative data from 563 participants, accounting for the fact that 2 pairs of studies shared participants but answered different research questions.^40–43^ Within this, 331 participants identified as male, 228 as female and 4 as transgender. Age was generally poorly reported but, from the available data, participants ranged in age from 19 to 66 years. Sample size varied from 12 to 195. Data collection methods included semi-structured interviews (*N* = 10), photo-elicitation interviews (*N* = 3), focus groups (*N* = 2), mobile phone diaries (*N* = 1), story elicitation interviews (*N* = 1) and life story interviews (*N* = 1). Relevant details of the included studies are outlined in supplementary Table 1.

### Quality assessment

Generally, quality ratings were high. All included studies chose appropriate qualitative methodology and produced valuable contributions. Studies were generally clear about recruitment and data collection and chose appropriate qualitative methodology. However, only two studies adequately considered the researchers’ relationship to participants and their own role, potential bias and influence.^23,42^ Table 3 presents the quality review ratings.

**Table 2:** The connectedness, hope, identity, meaning and empowerment (CHIME) processes of personal recovery^7^

First-order recovery process	Second-order recovery processes
(a) Connectedness	Peer support and support groups Relationships Support from others Being a part of the community
(b) Hope and optimism	Belief in possibility of recovery Motivation to change Hope-inspiring relationships Positive thinking and valuing success Having dreams and aspirations
(c) Identity	Dimensions of identity Rebuilding/redefining positive sense of identity Overcoming stigma
(d) Meaning	Meaning of mental health experiences Spirituality Quality of life Meaningful life and social roles Meaningful life and social goals Rebuilding of life
(e) Empowerment	Personal responsibility Control over life Focusing on strengths

**Table 3:** Quality review of the 16 included studies

Source	CASP criteria									
	Clear aims	Method	Design	Recruitment strategy	Data collection	Researcher bias	Ethical issues	Data analysis	Clear findings	Research value
Cabassa et al^44^	✓	✓	✓	✓	?	✗	✓	✓	✓	✓
Gopikuma et al^45^	?	✓	✓	✓	✓	✗	✓	✓	?	✓
Karadzhov^43^	✓	✓	✓	✓	✓	✓	✓	✓	✓	✓
Karadzhov^42^	✓	✓	✓	✓	✓	✗	✓	✓	✓	✓
Kerman & Sylvestre ^19^	✓	✓	✓	✓	✓	✗	✓	✓	✓	✓
Kirkpatrick & Byrne ^46^	?	✓	✓	?	✓	?	?	?	✓	✓
Kirst et al^47^	✓	✓	✓	✓	✓	✗	✓	✓	✓	✓
Macnaughton et al^48^	✓	✓	✓	✓	✓	✗	?	✓	✓	✓
Patterson et al^41^	✓	✓	✓	✓	✓	✗	✓	✓	✓	✓
Patterson et al^40^	✓	✓	✓	✓	✓	✗	✓	✓	✓	✓
Paul et al^49^	✓	✓	✓	✓	✓	?	✓	✓	✓	✓
Piat et al^50^	✓	✓	✓	✓	✓	✗	✓	✓	✓	✓
Reid et al^51^	✓	✓	✓	✓	✓	✗	✓	✓	✓	✓
Rhenter et al^52^	✓	✓	✓	✓	✓	?	✓	✓	✓	✓
Smith et al^53^	✓	✓	✓	✓	✓	?	✓	✓	✓	✓
Watson & Rollins^23^	✓	✓	✓	✓	✓	✓	✓	?	✓	✓

CASP, Critical Appraisal Skills Programme; ✓, yes; ✗, no; ?, not sure.

### Synthesis results

#### Recovery processes

Table 4 outlines the synthesis of data. Although most findings corresponded to the existing five first-order processes in the CHIME framework,^7^ two additional processes were identified: first, ‘security and stability’, which related to obtaining safe and stable housing, having a home versus a shelter, security in services and financial and food security. The second new first-order process, ‘dual recovery’, related to dual recovery from mental health issues and substance use. As with the original CHIME model, processes interacted and influenced one another. However, importantly, the data highlighted that ‘security and stability’ was foundational to the other recovery processes. Accordingly, SECURED was deemed a more fitting framework acronym. CHIME was transformed into SECURED through the addition of security and stability and dual recovery, as well as by the alteration of CHIME headings and their order: ‘connectedness’ was changed to ‘relationships and connectedness’; ‘hope’ to ‘encouragement and hope’; ‘identity’ to ‘constructing identity’; ‘meaning’ to ‘understanding and meaning’; and ‘empowerment’ remained the same.

**Table 4:** SECURED: mental health recovery processes for people with experience of homelessness

Recovery process	No. of studies represented *N* = 16
**1 Security and stability**	**13 (81%)**
** 1.1 Safe and stable housing**	9 (56%)
** 1.1.1 Having a home versus just shelter**	6 (38%)
** 1.1.2 Ability to look after physical health**	6 (38%)
** 1.2 Security in housing and services**	11 (69%)
** 1.3 Food and financial security**	5 (31%)
**2 Encouragement** and hope	**13 (81%)**
2.1 Belief in possibility of recovery	8 (50%)
2.2 Hope-inspiring relationships	6 (38%)
2.3 Having dreams and aspirations	8 (50%)
** 2.4 Hope for ‘normality’**	10 (63%)
**3 Constructing** identity	**13 (81%)**
3.1 Dimensions of identity	6 (38%)
3.2 **(Re)**building positive sense of identity; ‘**feeling like somebody’**	10 (63%)
3.3. Deconstructing **mental health and homelessness** stigma	6 (38%)
** 3.4 Drive to assume or reclaim a more powerful identity**	8 (50%)
** 3.5 Citizenship identity**	6 (38%)
**4 Understanding** and meaning	**16 (100%)**
4.1 Meaningful life and social roles and goals	10 (63%)
** 4.1.1 Engagement in meaningful activity**	8 (50%)
** 4.1.2 Helping others**	6 (38%)
4.2 Spirituality	4 (25%)
** 4.3 Understanding self and self-acceptance**	9 (56%)
4.4 Quality of life; **leaving ’survival mode’**	10 (63%)
**5 Relationships** and connectedness	**15 (94%)**
5.1 **Helpful** peer support and **social connections**	13 (81%)
** 5.2 Renegotiating relationships and social boundaries**	9 (56%)
** 5.3 Rebuilding relationships**	6 (38%)
5.4 Being a part of the community	8 (50%)
** 5.5 ‘Having someone in your corner’ in services**	10 (63%)
6 Empowerment	**14 (88%)**
6.1 Personal responsibility	4 (25%)
6.2 Control over life	9 (56%)
** 6.3 Self-efficacy in skills**	8 (50%)
** 6.4 Sense of ownership**	7 (44%)
**7 Dual recovery**	**9 (56%)**
** 7.1 Dual recovery from substance use and mental health difficulties**	9 (56%)
** 7.2 Support with substance use relapses**	4 (25%)

SECURED, security and stability; encouragement and hope; constructing identity; understanding and meaning; relationships and connectedness; and empowerment and dual recovery. CHIME, connectedness, hope, identity, meaning and empowerment. Themes shown in bold are additions or alterations to the CHIME framework. Total numbers for themes are also in bold.

The findings related to each of the seven key recovery processes that define the SECURED framework are outlined below.

**Security and stability.** A prevalent finding across many papers was security and stability in housing, services and basic needs as a necessary foundation for other recovery processes to take place. Stable and safe accommodation meant that people did not have to focus on survival and hypervigilance, which provided the opportunity to focus on recovery: ‘this is my home. I have a roof over my head and I can do the things I have to do and I can concentrate on my recovery’.^53^ Participants differentiated between a home and shelter. A home was described as a place with a sense of security, stability, privacy, ownership and control over their environment, in contrast to transitional housing or shelters, in which these things were often absent: ‘recovery was going from either living on the streets or transitioning from house to house to house or staying in unsafe environments, to having an apartment of my own … secure’.^23^ Having secure and safe housing, as well as food and financial security, offered opportunities for participants to look after their physical health, which contributed to improved overall well-being: ‘I sleep better, eat better, I feel secure.’^52^

**Encouragement and hope.** Optimism from the original CHIME ‘hope and optimism’ theme was removed because the *a priori* subthemes of ‘positive thinking and valuing success’ and ‘motivation to change’ from the original CHIME model were not found to be substantially endorsed in studies. Instead, encouragement from others was identified as being important in facilitating hope for this population, given the accumulated adversity many had faced over their lives. Relationships with peer workers inspired hope in some participants: ‘knowing that they’ve achieved what they achieved is like a light at the end of a tunnel’ .^51^ As with other processes, security and stability were often a prerequisite for hope, particularly housing. Security and reliability in services was important in fostering hope for some: ‘when I see everything happening according to the way they said it would, I was like this is the start of a new life for me’.^47^ Narratives of hope were often related to gaining a ‘normal life’: ‘I just wanted to have a normal life like everybody else.’^43^ In many studies hope also related to regaining control over one’s life, growing self-esteem and rebuilding and developing new relationships. Many participants also spoke about dreams and aspirations related to gaining employment and developing skills and relationships: ‘I am going to get my Grade 12 and I am going to take my nursing course.’^47^

**Constructing identity.** Building a positive sense of identity was an important recovery process, and was often related to building a sense of worth and personhood, in contrast to experiences of feeling dehumanised and devalued while homeless: ‘I see myself as a person of value now versus another nobody.’^51^ Being rehoused was again often a prerequisite for the reconstruction of a sense of self and identity, facilitated further by building connection with others, gaining self-acceptance and a sense of contribution or achievement. Deconstructing stigma as an original CHIME subtheme was expanded to incorporate stigma related to both homelessness and mental health, and often comprised deconstructing stigma on societal and internalised levels: ‘I’m in the healing process from a lot of stigma that society has put on me, a lot of self-stigma that I placed upon myself … I’m putting that stigma to rest.’^44^ Some participants spoke of a drive to assume or reclaim a more powerful identity, after feeling powerless while homeless: ‘I wanna live above what they [society] think I am.’^53^ Another new subtheme that was identified related to rebuilding a citizenship identity, having previously felt on the margins of, or excluded from, society: ‘What’s important to me is being a part of society. So, if I can’t work then I will volunteer.’^19^

**Understanding and meaning.** Understanding and meaning was the only first-order theme represented in all reviewed studies, and incorporated broad processes of finding understanding and meaning in the present, future and past. Participants discussed leaving ‘survival mode’^43^ by being housed and building food and financial security, enabling quality of life, and being able to ‘enjoy some nice things’.^48^ From this base, participants were then able to build a meaningful life through setting goals and finding direction: ‘[recovery] means that you always have a goal to try to do better for yourself’.^23^ Engaging in meaningful activity improved confidence, helped to regulate emotions, provided structure and a sense of progress and served as a distraction: ‘If you focus on something, it takes your depression mode away. It feels good because you’ve done/you’ve achieved something. It was a therapy.’^43^ Religion and spirituality also imparted meaning and facilitated a sense of security and hope during difficult times for some people: ‘God makes me stronger … that’s how I get my peace of mind by praying.’^44^

Understanding their mental health difficulties and the context in which they may have formed was described as a necessary next step following housing by some participants: ‘I first had to understand why I was feeling the way I was feeling.’^43^ Finding this understanding helped foster self-acceptance and compassion in others: ‘learn to at least like myself or not hate myself … trying to understand the different emotions about what happened in the past … and being able to see some worth in life’.^47^ This understanding and self-acceptance could be built upon by helping others, which facilitated finding meaning in their past, present and future: ‘your lived experience, which might have seemed like just a bad time or a mess, can be something productive for yourself and other people’,^51^ and seemed helpful for some participants in shifting their identity and social roles, from that of helped to helper: ‘I am an employee of the shelter that I used to stay in … that means something to me.’^19^

**Relationships and connectedness.** Social relationships were highlighted as being important across many studies. Opportunities to build positive new and helpful peer relationships in services and third-party organisations were beneficial: ‘my social group is much broader than it used to be … And that’s really what helped, [the feeling that] I’m not alone.’^51^ Sense of mutuality within this was useful: ‘a lot of trust because you’re with other people who have been in similar lived experience situations … you learn and grow together in that vital stage of starting out’.^51^ These new relationships were often a move away from unhelpful social relationships and environments experienced on the streets, in shelters and sometimes in their families. Participants discussed being able to ’set boundaries’ with those who did not have their best interests at heart: ‘I cut off a lot of people because … I am actually in the process of getting things together, but definitely these people were slowing me down.’^48^ Crucially, having secure, stable housing enabled participants to have more control in managing their social environment and relationships: ‘anybody wants to come through that door, it’s up to me to decide whether they get in or they don’t get in’.^46^ Being housed also offered the possibility of building or rebuilding relationships. For example, one participant discussed being able to invite his brother over to rebuild their relationship: ‘I have a place where I can receive him. That’s an important thing. So I can have a family again.’^47^

Another important part of connectedness was feeling as though there was ’someone in your corner’ in services – namely a professional who would provide respect, consistency, advocacy and support: ‘The one thing through all of that – my friends bailed, everybody bailed – I had that case worker … I knew that I had at least one person in my corner … I don’t think I would have been able to go to treatment the last time, the one that helped me stay clean, without them.’^19^ A sense of community belonging was also helpful and contributed to citizenship identity work: ‘I wasn’t a member of the community. I was what the community despised. The guy who hated … is now standing beside me to help save a tree.’^48^

**Empowerment.** Empowerment represents a crucial component of personal recovery in this population, given prevalent experiences of powerlessness while homeless. As with the original CHIME model, personal responsibility and having control over one’s life were endorsed subthemes. Having control was important for many people who had experienced a lack of control while homeless and in shelters. Control was interlinked with taking responsibility for decisions: ‘[I am now] able to make decisions, to take action on things … to build that support system so that when I feel as though I lose control, [I can] get back in control.’^51^ Two additional subthemes that supported the processes of responsibility and control were self-efficacy in skills and having a sense of ownership. Building skills in symptom management, aspects of daily living and beyond facilitated a sense of control and self-efficacy, which prompted personal responsibility: ‘To be the one who had the skills and executed them … I found that really, really empowering because I didn’t have to rely on anyone else to do it once I learned the skills.’^19^ A sense of ownership also represented control and autonomy for people who had long experienced deprivation: ‘I have my own appliances. That, to me, symbolises the autonomy [needed] to succeed.’^50^ For one participant, a sense of ownership also facilitated a greater sense of meaning in activity: ‘At [previous home], we had one, but it was a garden for them; it wasn’t a garden for me. Whereas this one, it’s really mine. I grow stuff for me.’^50^

**Dual recovery.** For many participants, recovery comprised dual recovery from substance use and mental health difficulties: ‘In my case, I think it’s all together.’^43^ Substance use issues had often developed prior to or while on the streets as a means of coping, survival and to ‘self-medicate’.^40^ Support for substance use issues was therefore an important part of mental health recovery for many participants. Secure and stable housing was again a prerequisite for supporting with substance use, with abstinence-based requirements for housing being unhelpful: ‘[you] don’t have to be sober … Getting me into that environment [permanent housing] became the number one thing to do first, and then out of that we were able to identify what the cause of the [substance use] problem was, treat the cause, and then go onto to stability in the other areas [of his life]’.^23^ Other participants emphasised the importance of control in this process: ‘they gave me the opportunity to make the decision [to quit using] at my own pace and my own time.’^23^

#### Challenges

Several challenges and barriers for personal recovery were identified from the data (see Table 5).

**Table 5:** Specific challenges and barriers in recovery for people with experience of homelessness

Challenges	No. of studies represented (*N* = 16)
1 Lack of security and safety	**11 (69%)**
1.1 Lack of secure and stable housing; ‘revolving doors’ of shelters and temporary housing	6 (38%)
1.2 Lack of safety; ’survival mode’ while homeless	6 (38%)
1.3 Ongoing poverty	2 (13%)
2 Hopelessness	**8 (50%)**
2.1 ‘Anti-recovery nature of homelessness’	4 (25%)
2.2 Idea of recovered life as unfamiliar/unclear due to lifelong adversity	5 (31%)
3 Negative identity experiences	**5 (31%)**
3.1 Ongoing stigma and dehumanisation	5 (31%)
4 Lack of meaning	**6 (38%)**
4.1 Boredom/lack of meaningful activity	6 (38%)
5 Disconnectedness	**10 (63%)**
5.1 Isolation on the streets and in shelters/apartments	7 (44%)
5.2 Unhelpful social relationships and influences	8 (50%)
5.3 Distrust in others	3 (19%)
6 Disempowerment	**11 (69%)**
6.1 Maltreatment in services	3 (19%)
6.2 Lack of control in shelters	7 (44%)
6.3 Discrimination and oppression	4 (25%)
7 Lack of support for cumulative and ongoing trauma	**4 (25%)**
8 Lack of support with substance misuse	**7 (44%)**

Total numbers for themes are in bold.

These challenges aligned with the SECURED themes but opposed the main recovery processes. It is important to recognise the challenges to personal recovery.^14^ However, because the current study aims are concerned with exploring processes supporting recovery, studies focusing solely on barriers were excluded, and this is therefore not a comprehensive list of challenges. Further description and illustrative quotes for the challenges identified in the included studies are detailed below.

### Lack of security and safety

Participants described being stuck in a cycle of the ‘revolving door of shelters, hotels, hospital stays and incarceration’.^40^ Without secure housing, participants remained in unsafe and often violent situations and needed to focus on survival rather than recovery: ‘I would roam the streets at night, and sleep in parks, parking lots … I ate from garbage cans.’^52^ Once housed, ongoing poverty prevented basic needs being met, presenting a barrier to other recovery processes: ‘poverty line is around $18,000, we make $10,000 [on disability income] … I have no choice’.^19^

### Hopelessness

Hope for recovery was described as difficult for some participants, due to lifelong adversity making recovery an elusive concept: ‘recovery is fearful to me. Because it’s the other side of life that I never experienced’;^43^ and others found that a lack of encouragement or support from services prevented hope: ‘nobody has shown it [hope] to me. No one has taken the time to … give me an inkling of hope’.^43^

### Negative identity experiences

Ongoing experiences of stigma and dehumanisation prevented positive identity changes: ‘“junkie”, “street person”, “you’re here again”’^19^; ‘they treat you like an animal’.^41^

### Lack of meaning

Without opportunities or support with engaging in meaningful activity once housed, some participants reported feeling directionless and without a sense of purpose, which exacerbated their mental health difficulties: ‘boredom. Just trying to kill the day and night away. It’s basically all you do every day, all day – just … kill time.’^42^

### Disconnectedness

Social isolation was an issue raised in many studies in regard to both homeless and housed participants. Without opportunities for social connection, participants could find themselves isolated when housed. Unhelpful relationships and social environments presented barriers to security, helpful connection and recovery from substance use: ‘there’s a ton of people there and I don’t feel comfortable. It’s full of lost souls’.^53^ Due to experiences of abuse and victimisation, some studies highlighted distrust of the system and other people as a barrier to building new relationships: ‘[with] what I’ve gone through, I find it hard to get close to people’.^40^

### Disempowerment

Experiences of disempowerment, through unhelpful services, a lack of control in shelters and discrimination and oppression, were common barriers to recovery. Homeless participants described discrimination regarding ‘half-arsed’ care and being viewed as a number in the shelter system rather than a person.^19^ Participants also experienced a lack of control in shelters due to rules: ‘we are not free in the shelter … The [shelter’s] schedule is one thing – waiting in the streets for three hours’;^52^ and in services taking uncollaborative approaches: ‘you don’t question that because you do not know better. It’s his job, he decides’.^19^

### Lack of support with cumulative and ongoing trauma

The cumulative trauma that is common in people with experience of homelessness often required therapeutic support that was difficult to access: ‘deep down, I’m still hurting like hell. I’m still screwed up … I need one-on-one counselling, but it’s hard to find’.^40^

### Lack of support with substance use issues

A lack of support with substance use issues was cited as a barrier to personal recovery from mental health difficulties: ‘I’m just going in circles. It feels like I’m trapped. Down here, I’ll always be an addict.’^41^

### Feedback from people with lived experience

Two people with lived experience of homelessness and mental health recovery consulted on the project and conveyed that the results corresponded with their experiences. Both relayed that the reality of services in the UK currently meant that they had experienced a lack of stable or secure support from services or housing for a long time, which exacerbated their mental health difficulties and thus emphasised the importance of early intervention with housing and mental health support. Their feedback did not alter the framework, but supported reflexivity and aided more nuanced discussion.

## Discussion

This systematic review aimed to synthesise the qualitative research on personal recovery in individuals with experience of homelessness, using a best fit framework synthesis to explore experiences and perspectives on personal recovery in this population, and to adapt the pre-existing CHIME model of personal recovery processes.^7^ Deductive analysis identified that the overarching CHIME themes were reflected in the included studies’ data. However, there was a greater emphasis on opportunities and support for the processes given the complex systemic challenges faced by this population, such as practical and material support. Inductive analysis identified security and stability (comprising secure and stable housing, having a home versus shelter and service, food and financial security) and dual recovery from substance use and mental health difficulties as additional key overarching processes. Inductive analysis also identified new subthemes within *a priori* CHIME themes, which are discussed below. There are challenges identified that oppose each of the recovery themes, in addition to difficulties with support for cumulative trauma. The addition of the two new overarching processes and the renaming and restructuring of the *a priori* CHIME headings transformed the CHIME framework into SECURED. A key finding is that security and stability was a necessary prerequisite for the other recovery processes in this population, and thus SECURED is suggested as a more fitting framework. SECURED maintains the original CHIME processes but restructures them to reflect security and stability as central to the other processes, and encompasses processes specific to this population. Taken together, the seven dimensions in the SECURED personal recovery framework provide a conceptual framework to support individualised conceptualisations of mental health personal recovery in people with experience of homelessness.

In addition to the new overarching themes of security and stability and dual recovery, *a priori* overarching themes were adjusted to incorporate subthemes specific to this population. Within encouragement and hope, this included hope for a ‘normal life’. Hope has been found to significantly correlate with measures of quality of life in individuals experiencing homelessness, emphasising the need for basic needs to be met as a foundational step towards hope.^54^ Within constructing identity, new themes included a drive for a more powerful identity, citizenship identity and deconstructing stigma regarding both mental health difficulties and homelessness. This links to research demonstrating the connection between perceptions of one’s social status and well-being.^55^ Regarding the theme of understanding and meaning, a greater emphasis was placed on engagement in meaningful activity once housed, social roles involving helping others and understanding one’s mental health difficulties to find meaning and self-acceptance in experiences. This aligns with previous research promoting the inclusion of self-acceptance in the CHIME framework.^56^ Relationships and connectedness were expanded to include enacting social boundaries regarding unhelpful social influences, rebuilding relationships, finding new helpful peer supports and social connections and having consistent support and advocacy from someone in services. Finally, the theme of empowerment included new additions of building self-efficacy in skills regarding management of mental health symptoms and daily living, as well as a sense of ownership, contributing to feelings of autonomy and empowerment. This aligns with the call for the addition of coping with difficulties to the CHIME framework.^14^

The concept of ontological security can help make sense of security and stability in housing, services, food and finances being a necessary prerequisite for the other recovery processes. Ontological security was originally coined in relation to mental health difficulties and is defined as ‘a sense of constancy in one’s social and material environment which, in turn, provides a secure platform for identity development and self-actualisation’.^56^ Homelessness represents a large rupture of ontological security.^57,58^ Security and stability as being conditional to the other recovery processes in the present review provides further support to the literature base detailing that stable housing underpins the development of ontological security.^57,58^ This could also be understood under the lens of Maslow’s^59^ hierarchy of needs theory, wherein basic needs must be addressed before the process of recovery or ‘self-actualisation’ can take place. This process is especially reflected in the theme understanding and meaning, wherein being housed and building food and financial security provided the quality of life necessary to leave survival mode and to be able to engage in the processes necessary for building self-understanding and meaningful activities. However, the findings of this review also highlight that housing alone is insufficient for recovery and that the other processes must follow.

Challenges were identified for all the recovery processes, including ongoing discrimination, stigma, lack of stable housing, trauma and poverty. This reflects the fact that recovery is not merely a personal process, and aligns with other research highlighting that social and systemic factors can either promote or inhibit recovery.^60^ Therefore, assisting recovery for people experiencing homelessness not only requires directly working with individuals, but also with services, social systems and communities.^14,60,61^

### Strengths and limitations

To the authors’ knowledge, this is the first systematic review of personal recovery experiences in the context of homelessness. The SECURED framework is potentially applicable to policymakers and intervention design, and also to further refinement as the literature grows. Expert-by-experience consultation is also a strength of this review. Limitations include the fact that the synthesis involved subjective interpretation and is derived from secondary data that are taken out of the context of data collection and not without their own bias.^62^ Furthermore, given that this is an emerging area of research, the included studies’ aims and contexts were broad, which may have limited comparability – for example, people with experience of sleeping in shelters versus those with experience of sleeping rough. Searching systemically, triangulating screening and quality assessment ratings with a second reviewer and a clear audit trial aimed to limit threats to the validity of findings.^63,64^ This review included only publications in English, and the studies were limited to the USA, Canada, Scotland, France and India. Notably, 9 of 16 of the included studies were conducted in Canada, which may limit the transferability of the model to other cultural and systemic contexts, particularly given differences in social services, healthcare provision and housing policy. As such, caution should be taken in generalising to broader populations, especially countries in the Global South, which were underrepresented (only one study was conducted in India).

### Implications for practice and research

This review responded to the recommendation to adapt the CHIME framework to specific populations,^14^ and thus SECURED provides a conceptual framework that could lend support to the growing drive to adopt a recovery-orientated approach within the homeless service system.^22^ The findings of this review also lend support to the Housing First approach to the homeless service system, wherein secure housing is prioritised before other issues are then addressed via intensive personalised support and case management.^65^ Importantly, housing is not contingent upon abstinence or treatment-first principles.^65^ In recent years, Housing First pilots and programmes have been expanding in the UK and across other parts of Europe.^66^ Nevertheless, findings highlight the fact that housing alone is not sufficient for personal recovery, and also support integrated service systems across social care, mental health and third sector services. Provision of an allocated case worker to oversee care across systems is helpful for trust and hope, and could contribute to ontological security. Research has highlighted improved outcomes in people experiencing mental health difficulties and homelessness who received intensive case management.^67^

People should be met with understanding that recovery may appear ambiguous in the context of their lives, and should be offered support and resources to explore different visions of a better life.^43^ Hope is a process that needs particular attention for this population, requiring regular encouragement,^47^ but this can prove difficult in systems with widespread underfunding, overwhelmed staff, stigma and other structural barriers.^43^ However, the current findings provide support for the usefulness of peer workers in supporting this process, by instilling hope and deconstructing dual stigma by contradicting internalised stereotypes.^51^ Opportunities to contribute to services and carry out peer work are beneficial for some individuals’ personal recovery, services and service users alike.

It is important that people are also offered prompt evidence-based mental health support, particularly regarding the cumulative trauma that many may have experienced.^52^ Findings from this review also highlight the importance of support with substance use issues, ensuring that individuals feel in control of their substance use recovery and are supported with relapses.^23^ Identification of individualised recovery goals should be supported across housing transitions, including support with employment.^48,51^ Peer and community groups can facilitate helpful social connections and a sense of community belonging.^51^ The current review findings also highlight the importance of supporting people to understand their mental health difficulties, find meaningful activity and build skills to support symptom management and daily living. This could be supported by recovery colleges,^51^ but also through community projects that may further aid with community belonging and rebuilding citizenship identity.

The processes identified in this review are suggested to be some common recovery processes from the existing literature. However, recovery sits within and is shaped by systemic, societal and personal experiences, and thus there should not be an expectation for recovery to appear in a certain way for any one individual.^68^ It is also important to avoid overemphasising individual or internally driven processes in personal recovery. For individuals experiencing homelessness, recovery is embedded within complex sociocultural and structural conditions. Structural inequities, including racism, sexism, ethnocentrism, stigma and systemic marginalisation, also intersect in ways that can significantly constrain or complicate recovery trajectories. An exclusive focus on individual-level change risks obscuring the broader social and political forces that shape whether, how and for whom recovery is possible. While this review focuses on important personal recovery processes, it is essential to recognise that these processes are embedded within wider sociopolitical systems, which play a critical role in shaping the possibilities and limitations of recovery.

Given that recovery is culturally influenced, future research is required into the relationship between mental health recovery and cultural norms (e.g. collectivism, family roles and spirituality) in the context of homelessness.^69^ This review incorporated challenges to the processes of personal recovery that were present in the included studies. However, this was not a comprehensive review of challenges to recovery. The aim of the review was to establish recovery processes, and thus studies focusing solely on barriers were not included. Future research synthesising the evidence base on the challenges to personal recovery is required. Furthermore, research into discriminatory and oppressive social structures and models of service provision and recovery would be useful.^70^ For example, there is promising research emerging into how Housing First models of care can be adapted to incorporate antiracism/anti-oppression practice.^71^

## Supporting information

Dring et al. supplementary material Dring et al. supplementary material

## Data Availability

Data sharing is not applicable to this article as no new data were created or analysed in this study.

## Supplementary material

The supplementary material is available online at https://doi.org/10.1192/bjo.2025.10851
