# Supplementary material for: Personal recovery in mental health difficulties in people with experience of homelessness: qualitative systematic review
Source: BJPsych Open. 2025 Nov 4;11(6):e265. doi: 10.1192/bjo.2025.10851 (PMC12641266; doi:10.1192/bjo.2025.10851)
Supplement: Dring et al. supplementary material [file S205647242510851Xsup001.docx]

| **Journal** | **Search Terms** | | | |
| --- | --- | --- | --- | --- |
|  | Homeless | Recovery | Mental Health Condition | Qualitative Method |
| Web of Science | homeless* OR unhoused OR "precariously housed" OR unsheltered OR "vulnerably housed" OR roofless OR "street person" OR "street people" OR "without home" OR "inadequate hous*" OR "insecure tenan*" OR hostel OR "couch surf*" OR ill-housed OR shelterless | recover* OR hope OR identity OR meaning OR empowerment OR connectedness | "mental health" OR "mental illness*" OR "mental disorder*" OR "mental disease*" OR "mental problem" OR "psychol* health" OR "psychol* illness*" OR "psychol* disorder" OR "psychol* problem" OR "psychiatr* health" OR "psychiatr* illness*" OR "Psychiatr* disorder" OR "psychiatr* problem" | ethnograph* OR qualitativ* OR "content analysis" OR "discourse analysis" OR interview OR "case study" OR "action research" OR "content analysis" OR "focus group" OR "narrative" OR "narratives" OR "thematic analysis" OR "mixed method" OR "multimethod study" OR "grounded theory" OR phenomenolog* OR observational |
| Psych INFO | homeless* OR unhoused OR precariously-housed OR unsheltered OR vulnerably-housed OR roofless OR street-person OR street-people OR without-home OR inadequate-hous* OR insecure-tenan* OR hostel OR vagrant OR couch-surf* OR ill-housed OR shelterless | recover* OR hope OR identity OR meaning OR empowerment OR connectedness | mental-health OR mental-illness* OR mental-disorder* OR mental-disease* OR mental problem OR psychol*-health OR psychol*-illness* OR psychol*-disorder OR psychol*-problem OR psychiatr*-health OR psychiatr*-illness* OR Psychiatr*-disorder OR psychiatr*-problem | ethnograph* OR qualitativ* OR content-analysis OR discourse-analysis OR interview OR case-study OR action-research OR content-analysis OR focus-group OR narrative OR narratives OR thematic-analysis OR mixed-method OR multimethod OR grounded-theory OR phenomenolog* OR observational |
| MEDLINE  &  PubMed | homeless* OR unhoused OR "precariously housed" OR unsheltered OR "vulnerably housed" OR roofless OR "street person" OR "street people" OR "without home" OR "inadequate hous*" OR "insecure tenan*" OR hostel OR "couch surf*" OR ill-housed OR shelterless | recover* OR hope OR identity OR meaning OR empowerment OR connectedness | "mental health" OR "mental illness*" OR "mental disorder*" OR "mental disease*" OR "mental problem" OR "psychol* health" OR "psychol* illness*" OR "psychol* disorder" OR "psychol* problem" OR "psychiatr* health" OR "psychiatr* illness*" OR "Psychiatr* disorder" OR "psychiatr* problem" | ethnograph* OR qualitativ* OR "content analysis" OR "discourse analysis" OR interview OR "case study" OR "action research" OR "content analysis" OR "focus group" OR "narrative" OR "narratives" OR "thematic analysis" OR "mixed method" OR "multimethod study" OR "grounded theory" OR phenomenolog* OR observational |
| Scopus | homeless* OR unhoused OR {precariously housed} OR unsheltered OR {vulnerably housed} OR roofless OR {street person} OR {street people} {without home} OR {inadequate housing} OR {insecure tenancy} OR hostel OR {couch surfing} OR ill-housed OR shelterless | recover* OR hope OR identity OR meaning OR empowerment OR connectedness | {mental health} OR {mental illness} OR {mental disorder} OR {mental illnesses} OR {mental disease} OR {mental problem} OR {psychological health} OR {psychological illness} OR {psychological disorder} OR {psychological problem} OR {psychiatric health} OR {psychiatric illness} OR {psychiatric disorder} OR psychiatr* OR {psychiatric problem} | ethnograph* OR qualitative OR {content analysis} OR {discourse analysis} OR interview OR {case study} OR {action research} OR {content analysis} OR {focus group} OR narrative* OR {thematic analysis} OR {mixed method} OR {multimethod studies} OR {grounded theory} |
| CINAHL | homeless* OR unhoused OR "precariously housed" OR unsheltered OR "vulnerably housed" OR roofless OR "street person" OR "street people" OR "without home" OR "inadequate hous*" OR "insecure tenan*" OR hostel OR "couch surf*" OR ill-housed OR shelterless | recover* OR hope OR identity OR meaning OR empowerment OR connectedness | "mental health" OR "mental illness*" OR "mental disorder*" OR "mental disease*" OR "mental problem" OR "psychol* health" OR "psychol* illness*" OR "psychol* disorder" OR "psychol* problem" OR "psychiatr* health" OR "psychiatr* illness*" OR "Psychiatr* disorder" OR "psychiatr* problem" | ethnograph* OR qualitativ* OR "content analysis" OR "discourse analysis" OR interview OR "case study" OR "action research" OR "content analysis" OR "focus group" OR "narrative" OR "narratives" OR "thematic analysis" OR "mixed method" OR "multimethod study" OR "grounded theory" OR phenomenolog* OR observational |
| Embase | homeless* OR unhoused OR "precariously housed" OR unsheltered OR "vulnerably housed" OR roofless OR "street person" OR "street people" OR "without home" OR "inadequate hous*" OR "insecure tenan*" OR hostel OR "couch surf*" OR ill-housed OR shelterless | recover* OR hope OR identity OR meaning OR empowerment OR connectedness | "mental health" OR "mental illness*" OR "mental disorder*" OR "mental disease*" OR "mental problem" OR "psychol* health" OR "psychol* illness*" OR "psychol* disorder" OR "psychol* problem" OR "psychiatr* health" OR "psychiatr* illness*" OR "Psychiatr* disorder" OR "psychiatr* problem" | ethnograph* OR qualitativ* OR "content analysis" OR "discourse analysis" OR interview OR "case study" OR "action research" OR "content analysis" OR "focus group" OR "narrative" OR "narratives" OR "thematic analysis" OR "mixed method" OR "multimethod study" OR "grounded theory" OR phenomenolog* OR observational |
| Social Services Abstracts | homeless* OR unhoused OR "precariously housed" OR unsheltered OR "vulnerably housed" OR roofless OR "street person" OR "street people" OR "without home" OR "inadequate hous*" OR "insecure tenan*" OR hostel OR "couch surf*" OR ill-housed OR shelterless | recover* OR hope OR identity OR meaning OR empowerment OR connectedness | "mental health" OR "mental illness*" OR "mental disorder*" OR "mental disease*" OR "mental problem" OR "psychol* health" OR "psychol* illness*" OR "psychol* disorder" OR "psychol* problem" OR "psychiatr* health" OR "psychiatr* illness*" OR "Psychiatr* disorder" OR "psychiatr* problem" | ethnograph* OR qualitativ* OR "content analysis" OR "discourse analysis" OR interview OR "case study" OR "action research" OR "content analysis" OR "focus group" OR "narrative" OR "narratives" OR "thematic analysis" OR "mixed method" OR "multimethod study" OR "grounded theory" OR phenomenolog* OR observational |
| ASSIA | homeless* OR unhoused OR "precariously housed" OR unsheltered OR "vulnerably housed" OR roofless OR "street person" OR "street people" OR "without home" OR "inadequate hous*" OR "insecure tenan*" OR hostel OR "couch surf*" OR ill-housed OR shelterless | recover* OR hope OR identity OR meaning OR empowerment OR connectedness | "mental health" OR "mental illness*" OR "mental disorder*" OR "mental disease*" OR "mental problem" OR "psychol* health" OR "psychol* illness*" OR "psychol* disorder" OR "psychol* problem" OR "psychiatr* health" OR "psychiatr* illness*" OR "Psychiatr* disorder" OR "psychiatr* problem" | ethnograph* OR qualitativ* OR "content analysis" OR "discourse analysis" OR interview OR "case study" OR "action research" OR "content analysis" OR "focus group" OR "narrative" OR "narratives" OR "thematic analysis" OR "mixed method" OR "multimethod study" OR "grounded theory" OR phenomenolog* OR observational |
